# Development of a classification scheme for disease-related enzyme information

**DOI:** 10.1186/1471-2105-12-329

**Published:** 2011-08-09

**Authors:** Carola Söhngen, Antje Chang, Dietmar Schomburg

**Affiliations:** 1 Technische Universität Braunschweig, Department of Bioinformatics and Biochemistry Langer Kamp 19 B, 38106 Braunschweig, Germany

## Abstract

**Background:**

BRENDA (**BR**aunschweig **EN**zyme **DA**tabase, http://www.brenda-enzymes.org) is a major resource for enzyme related information. First and foremost, it provides data which are manually curated from the primary literature. DRENDA (**D**isease **RE**lated **EN**zyme information **DA**tabase) complements BRENDA with a focus on the automatic search and categorization of enzyme and disease related information from title and abstracts of primary publications. In a two-step procedure DRENDA makes use of text mining and machine learning methods.

**Results:**

Currently enzyme and disease related references are biannually updated as part of the standard BRENDA update. 910,897 relations of EC-numbers and diseases were extracted from titles or abstracts and are included in the second release in 2010. The enzyme and disease entity recognition has been successfully enhanced by a further relation classification via machine learning. The classification step has been evaluated by a 5-fold cross validation and achieves an F1 score between 0.802 ± 0.032 and 0.738 ± 0.033 depending on the categories and pre-processing procedures. In the eventual DRENDA content every category reaches a classification specificity of at least 96.7% and a precision that ranges from 86-98% in the highest confidence level, and 64-83% for the smallest confidence level associated with higher recall.

**Conclusions:**

The DRENDA processing chain analyses PubMed, locates references with disease-related information on enzymes and categorises their focus according to the categories ***causal interaction***, ***therapeutic application***, ***diagnostic usage ***and ***ongoing research***. The categorisation gives an impression on the focus of the located references. Thus, the relation categorisation can facilitate orientation within the rapidly growing number of references with impact on diseases and enzymes. The DRENDA information is available as additional information in BRENDA.

## Background

Automatic bio-entity recognition and bio-entity relation classification has found a number of applications in the recent years [1,2]. On the one hand the work of manual expert information extraction is time-consuming and expensive and on the other hand computational power gets cheaper and the growth of publications in the biomedical field is tremendous. Thus applications in this area quickly gain more importance.

Some disease-related approaches concentrate on a rather precisely defined problem like the extraction of human kinase mutations [3]. The EnzyMiner routine [4] identifies PubMed abstracts that contain information on a mutation for an enzyme and tries to connect it to a disease. On the EnzyMiner website http://bioapps.sabanciuniv.edu/enzyminer/ disease and non-disease connected information on a very small number of enzymes (22) is available, mutation information on further enzymes has to be requested by an email form. Other information resources present information on all genes and proteins, their physical interactions and regulatory relationships like iHOP [5]http://www.ihop-net.org/UniPub/iHOP/ where information on enzymes and diseases can be found but is not in the very focus. MedEvi [6]http://www.ebi.ac.uk/Rebholz-srv/MedEvi/ is available through web-access as well and presents references and the particular evidence-sentence containing biomedical entities and semantic relations that are requested through a multi-term query.

For a number of years now the AMENDA and FRENDA databases are provided as a supplement to the manually annotated BRENDA enzyme data [7]. The data are extracted from literature abstracts using text-mining procedures.

Here we describe the development and contents of DRENDA, which is developed to complement the BRENDA knowledge system with information on diseases related to enzymes. BRENDA currently contains about 3 million qualitative and quantitative data on 4,624 active EC-numbers manually extracted from more than 100,000 references. The data cover multiple aspects associated with a descriptive view on enzymes such as classification and nomenclature, reaction and specificity, functional parameters, occurrence, enzyme structure, application, mutant information and engineered variants, stability, isolation and preparation. Although the number of annotated references increased by 30% since 2009 it is impossible to manually annotate all published references containing relevant information on enzymes. However, as the techniques and means of text mining and machine learning are improving it is possible to present automatically gained information, satisfying in quality and quantity.

The DRENDA text mining routine retrieves enzyme-related information on diseases in PubMed [8] by locating the incidence of enzymes and diseases in the literature. Enzymes represent the largest and most diverse group of all proteins. Due to their dominating role in metabolism and regulation, the malfunction of enzymes almost always leads to pathological processes in the organism. DRENDA provides information on the role of enzymes in diseases. Moreover all data are connected to a reference. A further classification of the enzyme role, e.g. the pharmacological or diagnostic value of an enzyme or its malfunction, is described in this paper.

The assignment of statements within a reference into the four categories *causal interaction, ongoing research*, *therapeutic application *and *diagnostic usage *allows to identify interesting biomedical aspects in the scientific literature of high information value. The categories were chosen so that it is quickly possible to get information on the role of enzymes in the context of diseases.

The Fabry disease is an inherited disorder of glycosphingolipid metabolism due to a deficiency of the enzyme alpha-galactosidase (EC 3.2.1.22), being an example for a *causal interaction *between an enzyme and a disease. The statement "*...a disease-specific therapeutic option - enzyme replacement therapy using recombinant human alpha-galactosidase has been recently introduced..*." [9] describes the *therapeutic application *in which the enzyme is the drug itself for the treatment of the disease.

More frequently the enzyme is used as the drug target as, for example, in therapeutic strategies that focus on the inhibition of enzymes of pathogenic viruses and bacteria. The title "The HIV-1 protease as a therapeutic target for AIDS" [10] is easily assigned to the category *therapeutic application*.

The title "Serum Alkaline Phosphatase Level as a Prognostic Tool in Colorectal Cancer..." [11] is an example for the assignment to the category *diagnostic usage*. The finding of abnormal activity of the isoenzymes (bone, liver) may indicate a broad range of pathological conditions like cirrhosis, hepatitis, liver tumours, drug intoxication, renal diseases, bone diseases and the presence of metastases.

The category *ongoing research *comprises references that contain statements indicating a preliminary relation between enzyme and disease but more research effort is necessary, e.g. indicated in the reference title "N-acetylgalactosaminyl transferase-3 is a potential new marker for non-small cell lung cancers." [12].

This publication focuses on the development and content of DRENDA which provides information on diseases and enzymes and their categorised relations.

## Methods

### Workflow and auxiliary means

**Figure 1:**
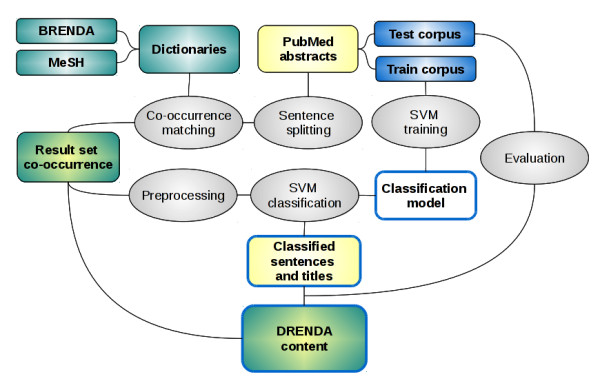
**A schematic illustration of the DRENDA work flow**. The BRENDA enzyme names and synonyms and the MeSH disease terms are used as dictionaries. The PubMed abstracts and titles are searched for co-occurring disease and enzyme entities. A test/train corpus was created for training an SVM and classifying the co-occurrence results according to the categories *causal interaction*, *therapeutic application*, *diagnostic usage *and *ongoing research*. The resulting entries are stored in the DRENDA database.

The DRENDA work flow is based on the disease mining procedures already included in BRENDA [13] but strongly enhanced inter alia by the added classification feature. The work flow (Figure 1) is implemented in Python and uses MySQL as database back-end. The first step is the initial sentence splitting (Figure 1, Sentence splitting) and search for the co-occurrence of enzyme and disease terms (Figure 1, Co-occurrence matching) within the title or an abstract sentence of the references enlisted in PubMed. The BRENDA enzyme names and synonyms, derived from manual literature annotation, comprise currently around 100,000 terms. This collection is certainly the largest available reference dictionary for the identification of enzyme entities (Figure 1, BRENDA).

The terms for disease recognition are taken from the **Me**dical **S**ubject **H**eadings (MeSH) [14]. The selected disease terms are a subset containing about 22,000 disease names and synonyms (Figure 1, MeSH). The titles and sentences, where at least one co-occurring enzyme and disease entity could be found (Figure 1, Result set co-occurrence) are the basis of the DRENDA content and stored as corpus for the subsequent classification.

### Relation classification

The classification is accomplished by machine learning methods. The basic classification unit is a sentence or title from PubMed abstracts where at least one enzyme and disease entity could be found (Figure 1, Result set co-occurrence). As they contain only a small number of words the problem is characterised by a sparse feature representation and offers the possibility to perform the classification via a **S**upport **V**ector **M**achine (SVM) that have proven to perform well on text classification tasks [15]. We have used SVMlight [16] as SVM implementation. In order to use an SVM for classification the availability of training and testing material is an essential requirement. For this purpose a corpus has been composed (Figure 1, Test corpus; Train corpus), which contains about 5,031 sentences and titles derived from PubMed abstracts. The selection out of all sentences and titles was made accordingly the following conditions: 2,500 have been randomly selected where at least one enzyme entity was identified. 2,000 have been randomly selected where at least one enzyme entity and one disease entity was identified. About 500 were randomly collected without any precondition from PubMed abstracts and titles. This corpus was manually annotated by an expert with biomedical background, for the identification of the presence of enzyme and disease entities and the assignment of all categories. If both entities are present within one sentence or title the relation was manually classified into the four categories of *causal interaction*, *therapeutic application*, *diagnostic usage *and *ongoing research*. In addition to the deployment as training and test data it is also used to evaluate the co-occurrence matches (Figure 1, Evaluation).

### Classification categories

#### Causal interaction

The crucial role of enzymes in catalysing metabolic reactions in organisms implies that malfunction, directly caused by a mutation in the coding gene or indirectly by the presence of inhibitors (e.g. side effects of drugs) or the absence of required cofactors, most often induces pathological conditions. The category *causal interaction *comprises references which describe such a relation between an enzyme and disease entity. The training/test corpus contains 1,382 sentences/titles which are annotated for the class *causal interaction*.

Example sentence for this category:

„*Chronic granulomatous disease (CGD) results from mutations of phagocyte NADPH oxidase.*“ [17]

#### Ongoing research

In a large number of publications a distinct interrelation of enzymatic function and the development or progress of a disease is presumed but not yet fully proven and further research investigation is needed. This instance describes the category *ongoing research*. 587 sentences of the training/test corpus are assigned to this category.

Example sentence for this category:

„*The prognostic significance of epidermal growth factor receptor (EGFR) expression in lung cancer and, more importantly, its ability to predict response to anti-EGFR therapies, are currently subjects of active research*.“ [18]

#### Diagnostic usage

In a clinical laboratory a multitude of parameters are examined for clarifying the presence of a pathological state or its severity. Many of these methods are based on the measurement of enzyme activities in the specimen.

Gamma-glutamyltransferase (EC 2.3.2.2) for instance, catalyses the transfer of a glutamyl residue from glutathione to an acceptor amino acid and its change in activity is used e.g. as an indicator of a liver dysfunction [19] which may be caused by excessive alcohol consumption. If an author states that an examination of an enzyme like the measurement of its activity, the test for its presence or the assay of its functional characteristic parameters are part of the diagnostic course of action, this reference is considered as included within the DRENDA category *diagnostic usage*. In the annotated training/test corpus 477 sentences/titles are in this category.

Example sentence for this category:

„*Prostate-specific antigen (PSA) is the most clinically useful tumour marker available today for the diagnosis and management of prostate cancer*.“ [20]

#### Therapeutic application

Given that enzymes play a major role in the development and progress of many diseases, they are also of relevance from the therapeutic point of view. On the one hand enzymes are considered as targets for drugs, on the other hand the enzyme itself may be the drug. If the enzyme is either a drug target or the drug agent for a mentioned disease, the corresponding reference is allocated to the category *therapeutic application*. The training/test corpus contains 366 sentences/titles corresponding to this category, about 62% of these sentences/titles describe cases where the enzyme is a drug target during the therapeutic intervention against the disease.

Example sentence for this category:

„*Indinavir sulfate is a human immunodeficiency virus type 1 (HIV-1) protease inhibitor indicated for treatment of HIV infection and AIDS in adults*.“ [21]

### Preprocessing and representation

In order to form a suitable input for SVM classification the set of titles and sentences, which were classified to contain a co-occurrence of an enzyme and disease, are prepared in a preprocessing step (Figure 1, Preprocessing).

First, terms that represent the entities "enzyme" and "disease" are removed (*removal*) or replaced (*replacement*) by a general term representing all enzyme respectively disease terms. This means that in the *removal *method all terms are deleted which are known to represent names and synonyms of enzymes or diseases without any substitution. In the *replacement *method the deleted names are replaced by a single generic term for all diseases and one generic term for all enzymes. Given that these two generic terms not have been present in the sentences and titles of the *result set co-occurrence *before their insertion during the preprocessing process.

The titles and sentences are represented as feature vectors within a document space [22]. The feature vector representation of a sentence or a title is performed via a calculation of the term frequency inverse document frequency *tf-idf*. The term frequency *tf_ij _*is the number of occurrences of the term *t_i _*in the particular sentences or title *d_j_*. The inverse document frequency *idf_i _*is calculated as(1)

where *D *is the overall number of sentences and titles and *d_it _*is the number of sentences and titles containing *t_i_*. *tf-idf *is the product of *tf_ij _*and *idf_i_*.(2)

Finally, all coordinates are divided by the length of the feature vector. Thereby each feature vector is converted to unit vector length, neutralizing the influence of the different sentence lengths.

### Classification of the co-occurrence results

As the next step of the described DRENDA work flow a classification of the co-occurrence results is performed. The test/training corpus has been randomly split into 4/5 training examples and 1/5 test examples. Every discrete classification category contains the same quantity of positive and negative annotated sentences/titles. The overall number of examples differs between each classification category due to the amount of positive assigned sentences/titles in each category. All sentences/titles which were positively annotated for one category are joined with the same amount of sentences/titles, negatively annotated for this category and containing at least one co-occurrence of an enzyme and disease entity.

A classification model (Figure 1, SVM training) is calculated and used to perform the classification of co-occurrence results (Figure 1, SVM classification). The classification results are finally distributed into a four level confidence system with descending classification precision and specificity from level 4 to 1.

### Cross validation

Before establishing the described DRENDA work flow (Figure 1) a five-fold cross-validation was performed to test the performance of the preprocessing steps and methods (*removal*; *replacement*) and the appropriate parameter choice for SVMlight. The overall ability of classification and assignment of sentences to the predefined categories was validated. In every category, *causal interaction*, *therapeutic application*, *diagnostic usage *and *ongoing research*, the same quantity of positive and negative annotated sentences/titles was chosen out of the training/test corpus and split randomly into five sets of equal size. Each individual set served once as a testing set while the other four formed a training set. Several calibrations of SVMlight parameters were tested as well as all four default kernel functions implemented in SVMlight (linear, polynomial, radial basis, sigmoid) were tested. This led to 2,688 distinct parameter combinations processed for each preprocessing method, *removal *and *replacement*. The classification of the co-occurrence results in the DRENDA work flow is processed once and not according to the cross validation scheme, due to processing time.

### Evaluation measures

In order to evaluate the correctness of the results derived from the co-occurrence based entity recognition (Figure 1, Result set co-occurrence) and the entity relation classification (Figure 1, Classified sentences and titles) steps in the DRENDA work flow some commonly used measures, including precision, recall, accuracy, specificity, F1 score and Matthews correlation coefficient (MCC), were calculated based on the test sets formed from the manually annotated corpus (Figure 1, Test corpus). The correct estimated presence of an enzyme and disease entity in the co-occurrence is counted as a true positive (*tp*), the correct estimated absence of one or both is counted as true negative (*tn*). If there are no co-occurring enzyme or disease entities or even one entity is missing but erroneously denoted as co-occurring it is considered as a false positive (*fp*) prospect. The case of co-occurring entities present which are not found by the routine is considered as false negative (*fn*). In the classification the correct assignment of a sentence to one of the classification categories is assessed as *tp*. The incorrect assignment to a classification category is assessed as *fp*. The correct neglected assignment to a classification category is assessed as *tn *and the incorrect neglected assignment as *fn*. The evaluation measures are defined as:(3)(4)(5)(6)(7)(8)(9)

In the five-fold cross-validation the values are also accompanied by the standard deviation.

In order to evaluate the benefit of a threshold variation to the predictive performance of the classification models receiver operating characteristic (ROC) curves have been plotted for the results of the five-fold cross-validation. In these ROC curves the average recall, or true positive rate, was plotted versus the false positive rate. The ROC plots and the calculation of the area under the curve (AUC) were performed by the use of the R package ROCR [23].

## Results and Discussion

### Enzyme and Disease related references

The content of DRENDA focuses on information on enzymes and ignores other proteins which may also be of biomedical relevance in a physiological and pathophysiological context. This selection is caused by the available extended dictionaries of enzyme names and on the close relationship of DRENDA as a text mining derived knowledge source which contributes to the expert annotated information of BRENDA [7].

Altogether 522,720 distinct references (table 1) were retrieved containing one or more co-occurring enzyme and disease. Within these references 4,061 different diseases are identified and assigned to 1,979 different EC numbers which leads to 112,805 distinct EC numbers/disease combinations. The number of DRENDA entries varies between enzyme classes (table 1). The hydrolases (EC class 3) are more frequently associated with pathological conditions and represent almost 50% of the DRENDA entries.

**Table 1:** A survey of the distribution of the co-occurrence derived results

Enzyme class (EC) name	EC	Diseases found with enzyme relation	EC numbers found with disease relation	Distinct combination of EC number and disease (a)	Distinct combination of EC number, disease and reference	Overall EC numbers in BRENDA (b)	Representation Quotient a/b
**Oxidoreductases**	1	2,646	429	23,214	159,215	1,393	0.76
**Transferases**	2	2,862	506	23,939	283,794	1,369	0.78
**Hydrolases**	3	3,439	737	55,036	406,831	1,523	1.64
**Lyases**	4	1,721	169	6,053	31,487	494	0.56
**Isomerases**	5	1,350	64	3,531	22,230	186	0.86
**Ligases**	6	712	74	1,578	7,340	161	0.45
	1-6	4,061	1,979	112,805	910,897	5,126	

a/b: The quotient of the combination of EC number and disease (a) and the overall EC numbers in BRENDA (b).

Notably peptidases (EC sub-class 3.4.) catalysing the cleavage of peptides and proteins form 53% of the DRENDA entries of hydrolases. They are the EC sub-class with the highest quantity of distinct EC numbers, containing with 41% a remarkable part of all hydrolases classified so far. Peptidases perform many essential functions within eukaryotic life e.g. in the blood coagulation cascade (thrombin, plasmin), the immunologic complement system (complement protease C1r, complement factor D), digestion of food proteins (pepsin, trypsin). Thus a deficiency of a peptidase may lead to manifold pathological processes. Their full functionality e.g. as virulence factors in bacteria (anthrax lethal factor endopeptidase) is also of pathogenic importance. In BRENDA about 20% of the inhibitor-related entries are connected to peptidases. This increased emergence indicates a high research interest especially in the medical field. Inhibition of a proteolytic enzyme is often a part of a potential therapeutic strategy. In comparison, other enzyme classes are not as strongly represented. The ligases (EC class 6) are rather infrequently connected to diseases (table 1). In BRENDA only 3% of the inhibitor-related entries are connected to ligases.

The results of the co-occurrence based disease and enzyme entity recognition were compared to the corresponding annotations of the test/train corpus. The co-occurrence related F1 score is up to 88.8% and shows an accuracy of 89.8%. The large collection of enzyme names in the reference dictionary is both an advantage and a disadvantage. Homonyms are one form of ambiguity. These are words that share the same spelling but have different meanings. They may lead to false positive matches. This originates from the use of general terms for enzymes, like the use of "lethal toxin" for anthrax lethal factor endopeptidase (EC 3.4.24.83), an enzyme that catalyses the hydrolysis of mitogen-activated protein kinase kinases and is secreted by virulent strains of the bacterium *Bacillus anthracis *as an enzymatic part of its exotoxin. Short acronyms in particular tend to be homonyms like "AAA", one of the synonyms for aryl-acylamidase (EC 3.5.1.13). "AAA" can have diverse meanings in a (bio-) medical article e.g. abdominal aortic aneurysm or American Ambulance Association. Where possible the dictionaries are optimized with respect to a deletion of homonyms and any ambiguous terms. The deletion of terms has to be considered with care because ignoring this terms also leads to a certain amount of missed entities (false negative) and thus the recall value decreases. Moreover there are other pitfalls in bio-entity recognition. There is a tendency in the literature to create new terms for enzyme disregarding the established systematic nomenclature. This increases the effort to keep dictionaries up-to-date with all used synonyms. Spelling mistakes also increase the difficulty in identifying entities.

### Classification performance check

**Figure 2:**
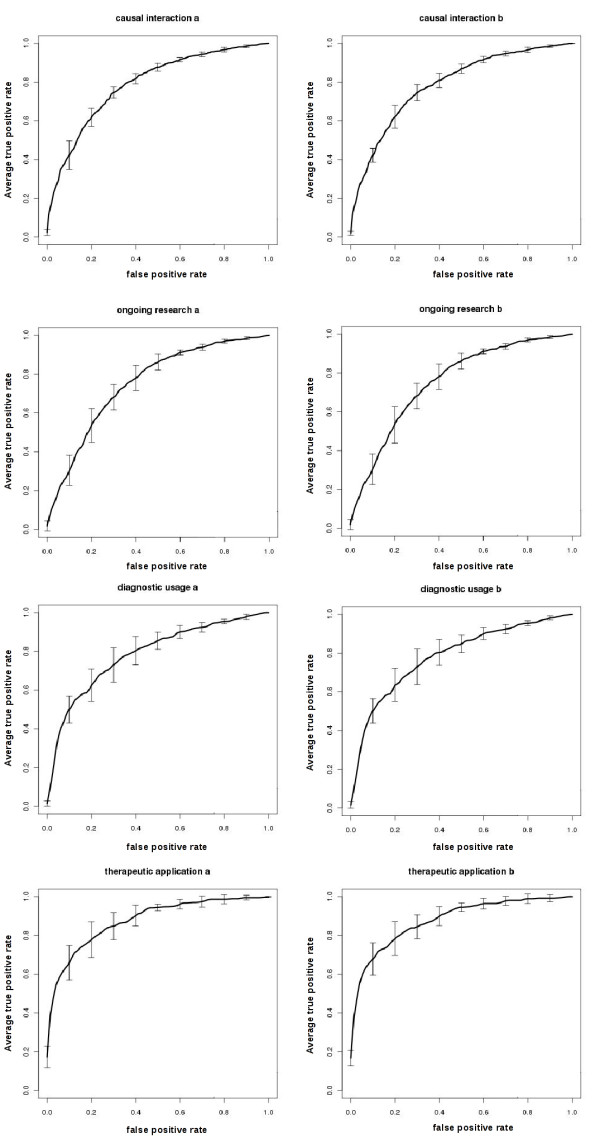
**Receiver operating characteristic (ROC) plots of the models, which achieved the maximal F1 scores**. The ROC plots shown belong to the models, which achieved the maximal F1 scores (table 2) in the five-fold cross-validation with either a *removal *(a) or *replacement *(b) preprocessing applied before the calculation of term weights. The ROC curves are vertical averaged (fixed false positive rates and averages of the corresponding true positive rates of each turn of the five-fold cross validation). In spite of decreasing standard deviation for larger numbers of available training sentences, the largest area under the curve (AUC) is achieved by classifiers for the category *therapeutic application*, which has least annotated sentences in the test/training corpus. See table 2 for the corresponding scalar AUC values of each plot.

The classification feature included in DRENDA was tested in a five-fold cross-validation. The F1 score for classification ranges between 0.802 ± 0.032 and 0.738 ± 0.033 (0.583 ± 0.076 and 0.395 ± 0.058 corresponding MCC) depending on the category and pre-processing procedures (table 2). One of the eminent observations of the cross-validation results is the rather small influence of the preprocessing methods of *removal *(table 2, a) and *replacement *(table 2, b) of terms representing the entities "enzyme" and "disease" on the F1 score and the AUC values (table 2) of the ROC plots in Figure 2.

**Table 2:** Maximal F1 scores achieved for the classification categories and the different preprocessing methods

Category	Preprocessing mode	F1 score	MCC	AUC
***therapeutic application***	a	0.802 ± 0.032	0.583 ± 0.076	0.878 ± 0.040
***ongoing research***	a	0.733 ± 0.024	0.395 ± 0.058	0.752 ± 0.037
***diagnostic usage***	a	0.738 ± 0.032	0.430 ± 0.074	0.784 ± 0.032
***causal interaction***	a	0.743 ± 0.009	0.429 ± 0.023	0.788 ± 0.020
***therapeutic application***	b	0.792 ± 0.016	0.548 ± 0.041	0.878 ± 0.038
***ongoing research***	b	0.744 ± 0.020	0.427 ± 0.051	0.752 ± 0.037
***diagnostic usage***	b	0.732 ± 0.022	0.412 ± 0.075	0.783 ± 0.033
***causal interaction***	b	0.742 ± 0.010	0.428 ± 0.024	0.786 ± 0.020

The category with the best F1 score is *therapeutic application *with 0.802 ± 0.032 for the *removal *preprocessing and 0.792 ± 0.016 for *replacement *preprocessing. This category shows also the maximum AUC values (0.878 ± 0.040 *removal*, 0.878 ± 0.038 *replacement*) and the maximum MCCs (0.583 ± 0.076 *removal*, 0.548 ± 0.041 *replacement*). The F1 scores of the categories *causal interaction *and *ongoing research *are comparable.

However, with an F1 score of 0.744 ± 0.020 and an MCC of 0.427 ± 0.051 for the *replacement *preprocessing, this method is more successful for the category *ongoing research *than the *removal *preprocessing (F1 score of 0.733 ± 0.024, and an MCC of 0.395 ± 0.058. In all other categories the *removal *of the terms representing the entities "enzyme" and "disease" achieved slightly better or equal results compared to the *replacement*.

In the five-fold cross-validation two preprocessing methods and 2,688 distinct parameter combinations were evaluated. Results for the models with the maximal achieved F1 scores, their corresponding Matthews Correlation Coefficient (MCC) and the area under the curve (AUC) of their receiver operating characteristic (ROC) plots shown in Figure 2. In the *removal *method (a) all terms representing the entities "enzyme" and "disease" were removed before the calculation of term weights, in the *replacement *method (b) these terms were replaced by a general term standing for all enzyme respectively disease terms.

A stop word *removal *with different combinations of high-frequency stop words was tested, but lowered the quality of the classification results (data not shown) so that it was decided not to remove stop words at all, apart from the described *removal *of terms representing enzyme and disease entities.

A word stemming stage was not included. This saves calculation time especially for processing a large corpus which is the result of the search for co-occurring enzyme and disease entities. Despite the omission of a word stemming processing step we achieve acceptable results regarding quality and quantity and could not observe any fatal influence on classification results.

### Interrelations of enzyme disease combinations

All sentences where an enzyme and a disease entity had been identified by the co-occurrence matching are classified by an SVM. The text corpus, consisting of the sentences and titles of the co-occurrence results (Figure 1, Result set co-occurrence), was preprocessed according to both methods *removal *and *replacement *of entity terms. For the classification of the co-occurrence corpus a reduced variation of the 57 best parameters of the 5-fold cross validation have been chosen. That comprises all parameter sets where a precision of at least 0.7 and an F1 score of at least 0.5 in one or more classification category. Each reference is assigned to one or more of the predefined enzyme and disease relation categories *causal interaction*, *therapeutic application*, *diagnostic usage *and *ongoing research*. The classification decision is accomplished by a binary SVM classification of each sentence or title. A reference can be assigned to none, one or more categories.

The classification results of the 910,897 distinct EC number/disease/reference combinations found by the co-occurrence matching are listed in table 3, table 4, table 5 and table 6 together with their quality parameters. The DRENDA classification result is distributed in four confidence levels. Higher confidence levels are characterized by higher precision but lower recall. As DRENDA is part of the BRENDA enzyme information system a high-quality result set is absolutely essential. For confidence level 4 the specificity is at least 96.7% or more and the precision is between 97.6% and 85.5% in all categories.

**Table 3:** Results and quality estimate of the category *causal interaction*

DRENDA confidence level	Distinct combination of EC number, disease and reference	Precision	Recall	Accuracy	Specificity	MCC
**4**	200,137	0.855	0.192	0.580	0.967	0.252
**3**	473,517	0.778	0.533	0.690	0.848	0.401
**2**	480,782	0.779	0.536	0.692	0.848	0.404
**1**	648,545	0.702	0.775	0.723	0.670	0.448

**Table 4:** Results and quality estimate of the category *ongoing research*

DRENDA confidence level	Distinct combination of EC number, disease and reference	Precision	Recall	Accuracy	Specificity	MCC
**4**	68,596	0.889	0.137	0.560	0.983	0.225
**3**	313,954	0.744	0.547	0.680	0.812	0.372
**2**	356,341	0.740	0.607	0.697	0.786	0.400
**1**	580,170	0.642	0.812	0.680	0.547	0.372

**Table 5:** Results and quality estimate of the category *therapeutic application*

DRENDA confidence level	Distinct combination of EC number, disease and reference	Precision	Recall	Accuracy	Specificity	MCC
**4**	158,143	0.976	0.548	0.767	0.986	0.594
**3**	182,532	0.938	0.616	0.788	0.959	0.612
**2**	345,421	0.855	0.808	0.836	0.863	0.672
**1**	422,601	0.831	0.877	0.849	0.822	0.700

**Table 6:** Results and quality estimate of the category *diagnostic usage*

DRENDA confidence level	Distinct combination of EC number, disease and reference	Precision	Recall	Accuracy	Specificity	MCC
**4**	193,632	0.939	0.326	0.653	0.979	0.403
**3**	354,083	0.833	0.632	0.753	0.874	0.521
**2**	401,714	0.795	0.695	0.758	0.821	0.520
**1**	454,540	0.764	0.716	0.747	0.779	0.496

Table 3 shows that 200,137 of the 910,897 combinations are assigned to the category *causal interaction *with a specificity of 96.7% in confidence level 4.

**Figure 3:**
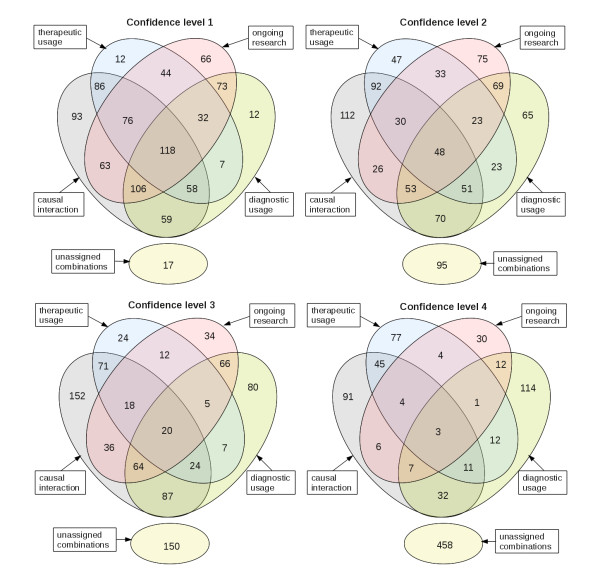
**The quota of intersection of classification categories (numbers × 10^3^, rounded)**. The overall amount of distinct EC, disease and PubMed reference combinations in the categories *causal interaction *(grey), *therapeutic application *(blue), *ongoing research *(pink) and *diagnostic usage *(green) in every DRENDA confidence level 1-4. The number of unassigned combinations is listed in the sets (yellow) at the bottom of each plot.

For each confidence level the number of matches in the category *causal interaction *is higher than in the other three categories (table 3, table 4, table 5 and table 6). A detailed view on the overlap of the four categories is given in Figure 3. A large subset of the categories *therapeutic application*, *diagnostic usage *and *ongoing research *overlaps with the set *causal interaction*. Throughout all four confidence levels about 50% of the results assigned to the particular category *therapeutic application*, *diagnostic usage *or *ongoing research *(Figure 3b-d) are also members of the category *causal interaction *(Figure 3).

This, of course, is expected as the fact that an enzyme causes a disease implies, that it often can be used in diagnosis or for therapy. For example the Gaucher's disease, a lysosomal storage disease which is caused by a hereditary deficiency of the enzyme glucosylceramidase (EC 3.2.1.45), is treated by a lifelong enzyme replacement therapy [24] and thus the enzyme is also the therapeutic agent. However, the proportion of entries belonging to all four categories decreases strongly with increasing confidence level. The selection in the higher confidence levels tends to prefer unequivocal statements and more ambiguous statements are rejected which reduces the number of entries intersecting all categories.

### Prostaglandin-endoperoxide synthase and its multiple relevance

Prostaglandin-endoperoxide synthase (EC 1.14.99.1), one of the most frequently retrieved enzymes, is registered with 15,103 entries associated to 832 diseases. It catalyses the initial step in the formation of prostaglandins, being the central enzyme in the biosynthetic pathway from arachidonic acid to prostaglandins.

The tissue distribution and physiological functions are depending on the isoform of prostaglandin-endoperoxide synthase. It is involved in platelet aggregation, gastric mucosa protection and renal electrolyte homeostasis [25], the inducible isoenzyme of prostaglandin-endoperoxide synthase is involved in inflammation processes and mitogenesis. It is expressed in response to stimuli like cytokines, growth factors, or hormones. Furthermore, the overexpression of the inducible isoenzyme is observed in pre-malignant and malignant neoplasms of the colon, liver, pancreas, breast, lung, bladder, skin, stomach, head and neck [26]. Prostaglandin-endoperoxidase synthase is a target of nonsteroidal anti-inflammatory drugs like Ibuprofen and Acetylsalicylic acid and is relevant in the treatment of mild to moderate pain, fever, inflammation processes and cardiovascular and rheumatic diseases. Hence it is not surprising that in each DRENDA confidence level it is one of the enzymes which ranges throughout in the top ten in the intersection set of all four classification categories.

Part of the training/test corpus was manually reannotated by a second expert with a background in biochemistry and enzymology in order to determine the inter-curator agreement. Reannotation was performed without knowledge of the annotation of the first curator (biomedical background). The amount of the inter-curator agreement ranged between 89.6% (*therapeutic application*) and 66.0% (*ongoing research*) with most of the differences occurring in the between *ongoing research *classification and one of the others. In many cases the difference occurred in the assessment whether statement made by the authors is strong enough to put it into one of the defined categories *causal interactions, diagnostic or therapeutic *or whether it is additional research is needed.

### Comparison with DrugBank

The DrugBank [27] database supplies information on drugs and their related drug targets. We compared our results of the category *therapeutic application *with the entries of DrugBank version 2.5. The 39,023 entries for drug targets in DrugBank consist of 9,713 distinct terms of which 1,187 could be identified as an enzyme entity and associated with an EC number. From the DrugBank text entry indications we could identify 264 disease entities representing about 6% of the distinct disease entities included in DRENDA. A comparison showed that between 72.8% and 76.4% of the combinations of EC number, disease and PubMed reference in DRENDA are confirmed by DrugBank (table 7), depending on the confidence level.

**Table 7:** The DrugBank entries for drug targets in comparison with the entries of DRENDA

DRENDA confidence level	EC numbers in DRENDA	DRENDA/DrugBank agreement	EC number, disease and PubMed reference combination	DRENDA/DrugBank agreement	DRENDA/DrugBank agreement with consideration of diseases
**1**	1,622	863 (53.2%)	422,601	307,477 (72.8%)	18,834 (4.5%)
**2**	1,516	831 (54.8%)	345,421	251,460 (72.8%)	17,018 (4.9%)
**3**	1,227	710 (57.9%)	182,532	137,701 (75.4%)	12,466 (6.8%)
**4**	1,183	694 (58.7%)	158,143	120,760 (76.4%)	11,448 (7.2%)

The entries of DRENDA which have are assigned to the category *therapeutic application *put in relation to DrugBank entries. The amount of EC numbers and combinations of EC number, disease and PubMed reference are listed together with the quantity of coinciding DrugBank entries for drug target enzymes with and without consideration of matching disease.

### Accession to DRENDA

**Figure 4:**
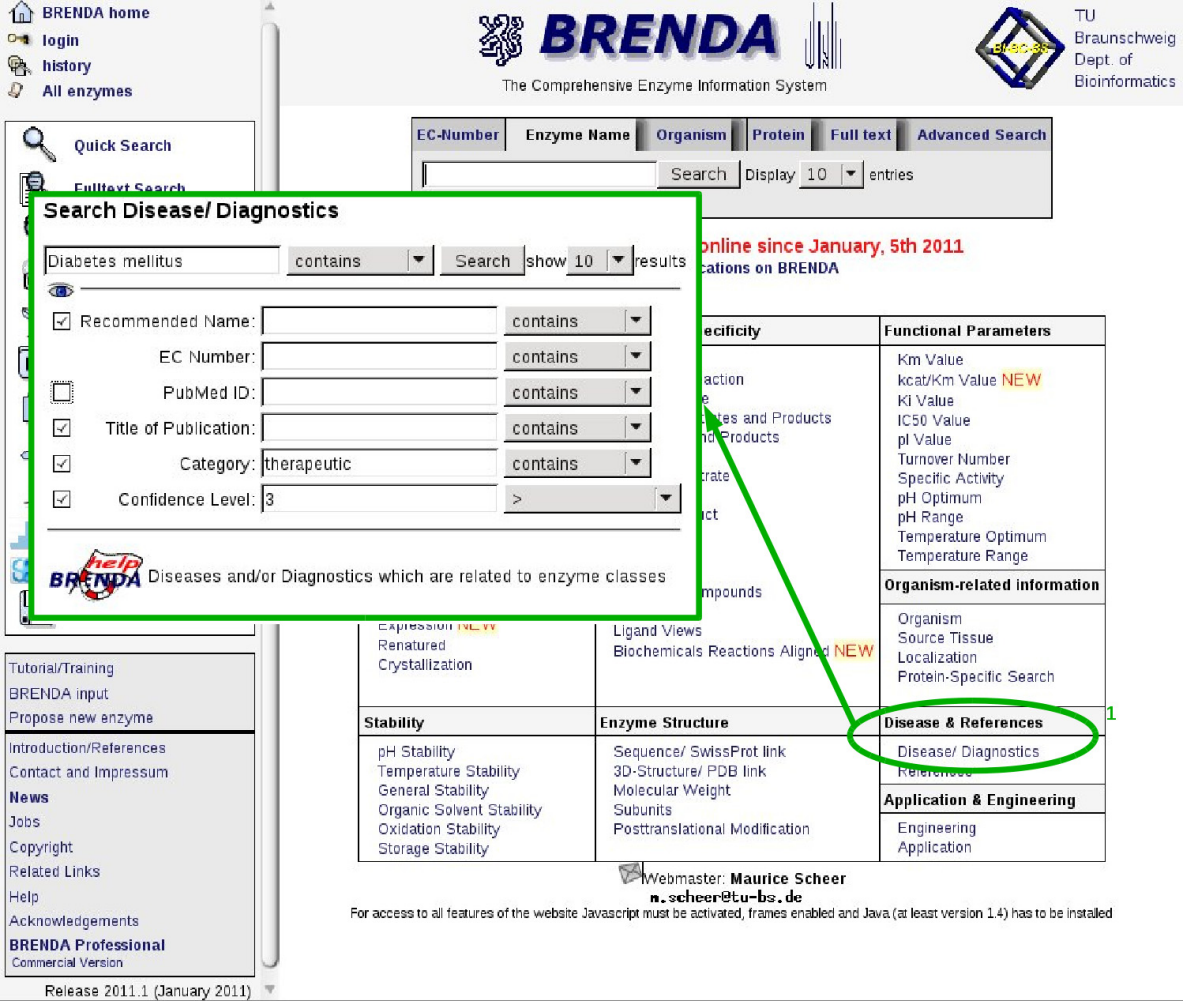
**Screen shot of the BRENDA web portal entry with a view on the DRENDA query form**.

**Figure 5:**
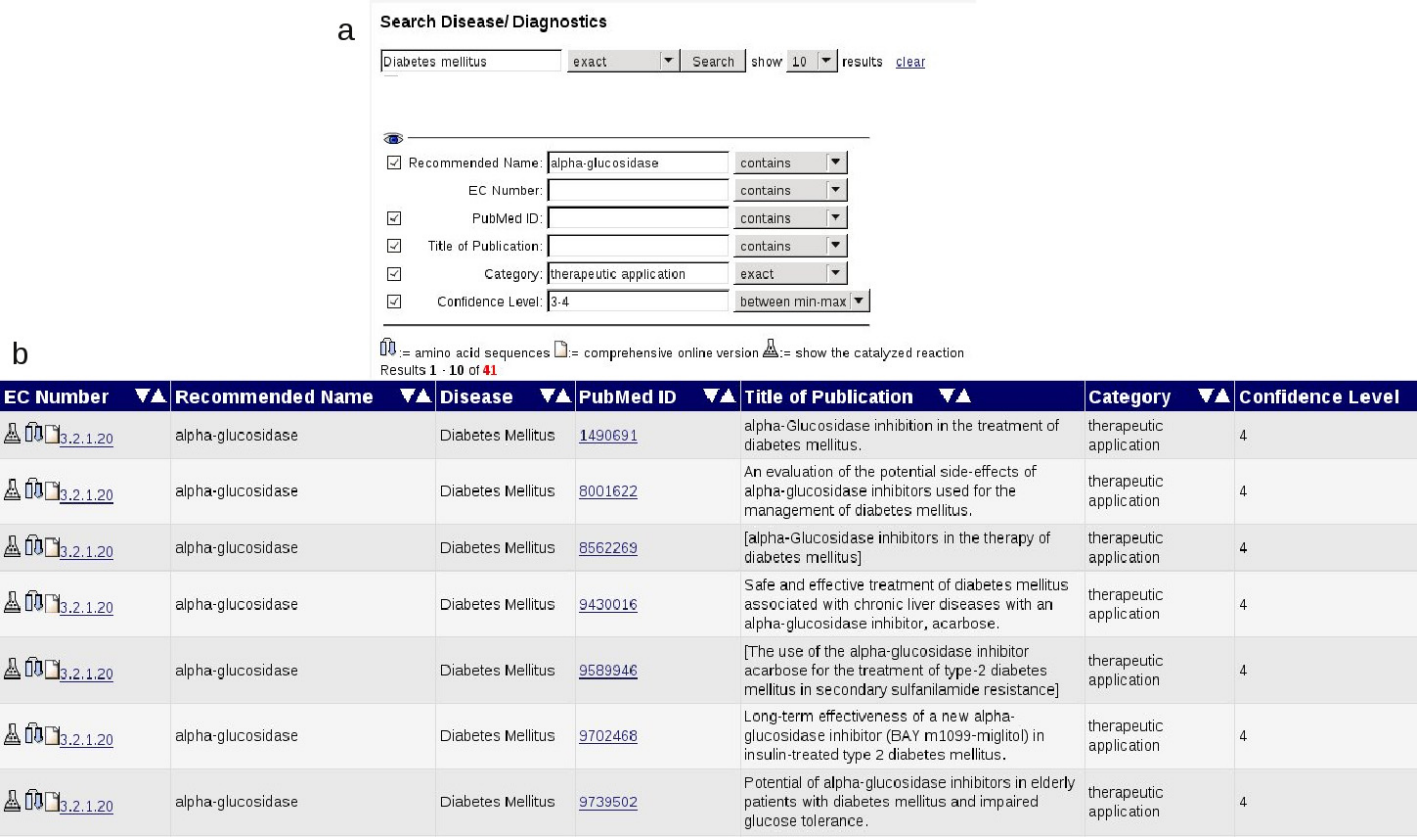
**Access to the DRENDA data**. The query form (a) provides several fields for entering search pattern information. The fields can be combined arbitrarily for a refinement of the query and meet individual requirements. As an example, a part of the query result table (b) for "Diabetes mellitus" as requested disease and all entries assigned to the category *therapeutic application *with a DRENDA confidence level of 3 and 4.

The DRENDA disease and enzyme related data are available via the BRENDA web portal http://www.brenda-enzymes.org in the section "Disease/Diagnostics" (Figure 4). The DRENDA data are fully searchable via a query form (Figure 5a). The fields provided allow searching a full or partial name of a disease, an enzyme, an EC number or the title of a publication. These fields can individually be combined for an optimal refinement of the query. By the selection of the check boxes of the fields "Category" or "Confidence Level" the classification results of the retrieved references are visible. By submitting the query a result table is created, according to the requested information (Figure 5b). Every entry contains links to the BRENDA flat file view of the enzyme, a search for amino acid sequences of the enzyme, a view on the catalysed reaction and a link to the abstract of the reference to PubMed. Thus DRENDA results are effectively connected to gain close information about the enzyme from the BRENDA information system.

## Conclusions

DRENDA provides broad information on enzymes and diseases arranged according to EC number and disease name. A first version of DRENDA is accessible via the BRENDA web portal http://www.brenda-enzymes.org. The DRENDA information collection contains the co-occurrence based enzyme/disease entries and the accession to the additional information on the type of relation of the enzyme and the disease. The statements and conclusions within a reference on the relation of an enzyme and a disease are assigned to the categories *causal interaction, ongoing research*, *therapeutic application *and *diagnostic usage*. The DRENDA content is biannually updated in the course of a new BRENDA release. Thus DRENDA covers interesting biomedical aspects like the pharmacological or diagnostic value of an enzyme in connection to a disease.

## Authors' contributions

CS designed the study, implemented the algorithm, curated the data set, performed the analyses and drafted the manuscript with AC. AC curated the data set and drafted the manuscript with CS. DS conceived and oversaw the project, participated in its design and coordination, and helped to draft the manuscript. All authors read and approved the final manuscript.
